# Specialist pollinators deplete pollen in the spring ephemeral wildflower *Claytonia virginica*

**DOI:** 10.1002/ece3.2252

**Published:** 2016-06-28

**Authors:** Alison J. Parker, Neal M. Williams, James D. Thomson

**Affiliations:** ^1^ University of Toronto25 Harbord StreetTorontoONM5S 3G5Canada; ^2^ University of California‐Davis380 Briggs HallOne Shields Ave.DavisCalifornia95616

**Keywords:** Fly pollination, oligolectic bees, plant–pollinator specialization and generalization, pollen collectors, pollination

## Abstract

Pollinators that collect pollen – and specifically, pollen‐specialist bees – are often considered to be the best pollinators of a (host) plant. Although pollen collectors and pollen specialists often benefit host plants, especially in the pollen that they deliver (their pollination “effectiveness”), they can also exact substantial costs because they are motivated to collect as much pollen as possible, reducing the proportion of pollen removed that is subsequently delivered to stigmas (their pollination “efficiency”). From the plant perspective, pollen grains that do not pollinate conspecific stigmas are “wasted”, and potentially costly. We measured costs and benefits of nectar‐collecting, pollen‐collecting, and pollen‐specialist pollinator visitation to the spring ephemeral *Claytonia virginica*. Visits by the pollen‐specialist bee *Andrena erigeniae* depleted pollen quickly and thoroughly. Although all pollinators delivered roughly the same number of grains, the pollen specialist contributed most to *C. virginica* pollen delivery because of high visitation rates. However, the pollen specialist also removed a large number of grains; this removal may be especially costly because it resulted in the depletion of pollen grains in *C. virginica* populations. While *C. virginica* appears to rely on pollen transfer by the pollen specialist in these populations, nectar‐collecting visitors could provide the same benefit at a lower cost if their visitation rates increased. Pollen depletion affects a pollinator's value to plants, but is frequently overlooked. If they lower the effectiveness of future floral visitors, visits by *A. erigeniae* females to *C. virginica* may be more detrimental than beneficial compared to other pollinators and may, in some circumstances, reduce plant fitness rather than increase it. Therefore, *A. erigeniae* and *C. virginica* may vary in their degree of mutualism depending on the ecological context.

## Introduction

What makes a good pollinator? Animal‐mediated pollination requires removal of pollen from anthers, transport, and deposition onto receptive stigmas. In generalized pollination systems, one plant species is often visited by a variety of potentially pollinating species with diverse attributes and characteristics that may affect the quality and quantity of pollen transferred through different steps in the process. Plant individuals benefit most from animal pollination when pollen transfer is efficient, that is, when most of the pollen grains that are produced are transferred to receptive, compatible stigmas. However, plant individuals pay a cost for pollination service: not all pollen that is removed is successfully deposited, resulting in “wasted” pollen.

The cost of wasted grains to plants will vary depending on the pollination context. Wasted pollen will be more costly when the supply of pollen is limited than when it is abundant because pollen will not be available for future pollinators, therefore potentially resulting in fewer mating opportunities for the pollen‐donating plant (Thomson 2003). Thus, pollinator visits have higher relative costs when they result in “pollen depletion,” a reduction in the pollen standing crop remaining in an individual flower to the point that future visitors are not able to remove the amount that they would otherwise. Pollen depletion may affect an individual plant's siring success because of lost opportunities for pollen export by future flower visitors (Hargreaves et al. 2009, 2010). In pollen‐limited populations, pollen depletion at the population level may also decrease pollen transfer and pollen receipt in the population as a whole (Hargreaves et al. 2010). This differential depletion‐related cost creates the potential for flower visitors to vary in their degree of mutualism, not just through how effectively they deposit pollen, but through the proportion of pollen removed that is delivered and their depletion of pollen resources.

Measuring the costs of wasted pollen is difficult; few studies are quantitative and thorough. Several studies have compared the amount of pollen removed by different pollinators by measuring the number of grains that each floral visitor removes in a single visit (“single‐visit pollen removal”, e.g., Larsson 2005; Sahli and Conner 2007; Young et al. 2007; Zych et al. 2013). However, these studies do not assess the cost of pollen removal because they do not consider pollen depletion, which has rarely been measured empirically. We found only four examples of pollen depletion measurements in the literature, and these studies measured population pollen depletion for only 1 day (Wilson and Thomson 1991; Minckley et al. 1994; Raine et al. 2006) or measured pollen depletion in an agricultural setting supplemented by commercially produced pollinators (Stanghellini et al. 2002).

Predicting the effects of pollen depletion on pollen delivery has inspired a set of theoretical “pollen depletion models” (Harder and Thomson 1989; Thomson and Thomson 1992; Castellanos et al. 2003; Thomson 2003). These models use basic empirical data such as the visitation rate, single‐visit pollen removal, and the number of grains delivered in a single visit (“single‐visit pollen deposition”) to calculate pollen depletion and hypothetical pollen delivery under a scenario of concurrent visitation by multiple species of flower visitors; they demonstrate that a flower visitor that wastes pollen may reduce pollen delivery by depleting pollen and impacting the pollen transfer of future flower visitors. To evaluate these theoretical results, empirical measurement in natural systems is an important next step.

The cost of pollen depletion may also affect the evolution of floral traits. The number of pollen grains a floral visitor can remove in a single visit can be limited by the rate at which pollen is made available for removal (the “pollen presentation schedule”). Selection may act to separate pollen into packages or dispense pollen from these packages over time – that is, through multiple anthers and the dehiscence of these anthers over time –because limiting pollen collection could reduce pollen depletion and mitigate the cost of wasted pollen to plants (Harder and Thomson 1989). Therefore, the rate of anther dehiscence in plant individuals and species may reflect selection pressure exerted by flower visitors that waste pollen (e.g., Li et al. 2014).

The foraging biology of flower visitors can affect their importance as pollinators. Flower visitors can include primarily nectar‐collecting species (e.g., most flies, butterflies, male bees, birds, moths, and bats), those that collect both nectar and pollen (e.g., most female bees), and sometimes one or more flower visitors that collect pollen from only that plant species and close relatives (e.g., pollen‐specialist [oligolectic] bees). Some researchers consider bees generally, and pollen‐specialist bees in particular, to be the most important pollinators to a (host) plant (Vogel and Machado 1991; Freitas and Sazima 2003; Hoffmann and Kwak 2005; McIntosh 2005); bees forage efficiently and systematically (Harder 1990; Chittka et al. 1997), and pollen‐specialist bees by definition focus their pollen foraging effort primarily on their host plant and may be adapted to be better able to handle host plant flowers quickly (Strickler 1979; Thorp 1979; Laverty and Plowright 1988; Cane and Payne 1993; Schlindwein and Wittmann 1997; Minckley et al. 1999; Müller and Bansac 2004; Moeller and Geber 2005; Raine and Chittka 2006, 2008). However, these factors may impose costs to flowering plants because female bees are selected to efficiently deliver pollen from anthers to their larvae, and from a plant perspective, this pollen is wasted. Pollen‐specialist bees have been shown to collect more pollen per foraging effort than pollen‐generalist bees (Strickler 1979; Laverty and Plowright 1988; Cane and Payne 1993). Moreover, bees can learn sophisticated behaviors for exploiting plant resources, like foraging preferentially on a particular flower gender (e.g., Ågren et al. 1986; Bierzychudek 1987; Ashman and Stanton 1991; Eckhart 1991; Wilson and Thomson 1991; Delph and Lively 1992; Ashman 2000); pollen‐specialist bees appear likely to conduct these behaviors.

When pollen‐collecting bee species – and pollen‐specialist bee species in particular – have evolved to maximize their collection effort, it follows that high visitation by these bees may deplete the supply of pollen in plant individuals and populations. Pollen‐collecting bee species (both generalist and pollen specialist) have been shown to remove a great deal more pollen than primarily nectar‐collecting visitors in some systems (e.g., Larsson 2005). More pollen removal does not always lead to more pollen delivery. “Cheater” floral visitors can remove floral resources (“consumptive emasculation” of pollen) and not contribute to pollen delivery (Hargreaves et al. 2009, 2010; Padyšáková et al. 2013). Even when some pollen is delivered, bees that remove more pollen often deposit a smaller percentage of those pollen grains on subsequent flower visits (Harder and Thomson 1989). If pollen‐collecting and pollen‐specialist bee species are causing pollen depletion and pollen is limited in a plant population, visits by these species may not be increasing pollen export and delivery and may instead be reducing plant mating opportunities and lowering the fitness of pollen exporting plants (male fitness). If pollen is limited in a plant population, pollen depletion may also be lowering the fitness of pollen receiving plants (female fitness) (Wilson and Thomson 1991; Hargreaves et al. 2010). As a result, pollen‐collecting and pollen‐specialist bees may vary in their degree of mutualism, despite acting as pollinators by transferring pollen grains.

Here, we compare the costs and contributions of different pollinator groups, including primarily nectar‐collecting individuals, primarily pollen‐collecting individuals, and pollen specialists, to the pollination of a spring ephemeral wildflower. We link pollen‐specialist removal to substantial pollen depletion in plant populations, providing a more comprehensive view of the cost of pollen‐specialist visitation.

## Materials and Methods

**Figure 1:**
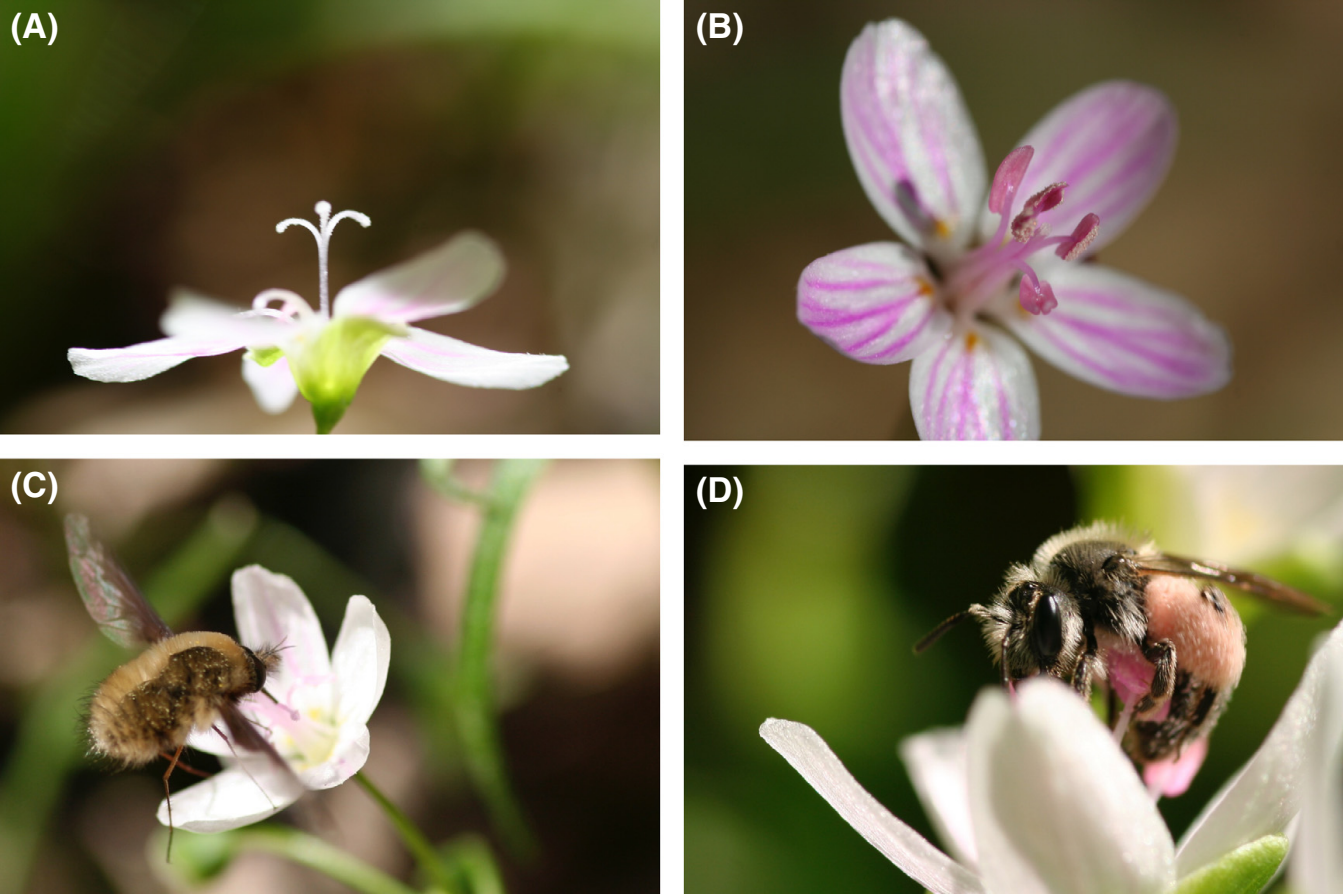
Photographs of the *Claytonia virginica* pollination system. (A) A *C. virginica* female‐phase flower. (B) A *C. virginica* male‐phase flower. (C) The bee‐fly *Bombylius major* visiting *C. virginica*. (D) The oligolectic bee *Andrena erigeniae* visiting *C. virginica*.

*Claytonia virginica* L., “Spring Beauty” (Portulacaceae) is a spring ephemeral wildflower native to North American eastern woodlands, ranging from Georgia to Ontario and from the East Coast to Kansas and Nebraska. Flowers are protandrous; pollen and nectar are offered on the first day, in the male phase, and only nectar is produced in the female phase (Fig. 1). On the second day, as the flower opens the three lobes of the stigma unfold, indicating that the stigma is receptive (Motten et al. 1981). The nectar in pollinator‐excluded second day flowers contained twice the sugar of first day flowers, indicating that nectar production rates of male‐ and female‐phase flowers are approximately equal (Motten et al. 1981). *Claytonia virginica* is self‐compatible but not self‐pollinating (Motten et al. 1981), and self‐pollinated flowers produce fewer seeds than outcrossed flowers (Schemske 1977). The flowers are visited by a variety of insects, among them the pollen‐specialist solitary bee *Andrena erigeniae*, which collects pollen exclusively from *C. virginica* and the closely related *Claytonia carolinana* (Fig. 1, Davis and LaBerge 1975). The geographic range and phenology of *A. erigeniae* match that of *C. virginica* (Davis and LaBerge 1975). A number of generalist insect species also visit, collecting pollen, nectar, or both. The most frequent generalists are the bee‐fly *Bombylius major*, which does not actively collect pollen, and generalist bees in the genera *Lasioglossum*,* Ceratina*, and *Hylaeus*, which collect both pollen and nectar from *C. virginica*.

Male‐phase *C. virginica* flowers have five anthers with two locules each, therefore separating pollen into 10 “packages” that dehisce one at a time. Anthers usually dehisce during the first hour or two of the flower's opening; dehiscence occurs faster in warmer temperatures.

*Claytonia virginica* has been shown (in pollen supplementation experiments) to exhibit varying degrees of pollen limitation in some Indiana and Pennsylvania populations (C. Lin, pers. comm., Williams and Winfree 2013) and was not pollen‐limited in a few North Carolina populations (Motten et al. 1981).

We collected data in a number of sites in suburban Philadelphia, Maryland, and North Carolina in Spring 2009 and Spring 2010. The majority of the data were collected in 2009 on five populations of *C. virginica* in suburban Philadelphia; these populations were located on the grounds of Lankenau General Hospital, Andorra Woods, Ridley Creek State Park, and two private woodlots. All pollen depletion data are from these Pennsylvania populations. To increase our sample sizes for pollen removal and deposition, we included additional data collected in Spring 2010 at Mason Farm Biological Reserve in Chapel Hill, North Carolina and Patuxent National Wildlife Refuge in Laurel, Maryland.

We collected data during a “daily activity window” during which *C. virginica* flowers were donating and receiving pollen; data collection began with the start of anthesis in the *C. virginica* population and when female‐phase flowers in the population were open and ended when female‐phase flowers in the population were closing. This period generally coincided with when insect activity on *C. virginica* began and ended. In early spring, these events were highly variable due to weather. The daily activity window (insect activity, the start of anthesis, and the opening of female‐phase flowers) began as early as 8:45 am on warm and sunny days, but on cool days or after rain this often did not occur until late morning or early afternoon. Data collection continued until female‐phase flowers in the population began to close or insect visitation ended, which on warm days occurred as early as noon. Usually, the daily activity window lasted only 2–4 h.

### Single‐visit pollen removal

We measured single‐visit removal by each of the three pollinator groups as the difference between the number of pollen grains remaining within the anthers of a flower after a single visit and the number of grains in a sample of unvisited control flowers. Before the daily activity window began (before the start of anthesis), we covered flowers with cages with mesh small enough to prevent visitor entry but allow for air flow. At the onset of pollinator activity and throughout the course of the day, we uncovered these unvisited flowers and allowed a single visit from a free foraging insect. All visited and control flowers had anthers that were totally dehisced. Many single visits were conducted in situ; however, in order to encourage visitation and increase sample sizes, we often inserted our prepared flowers into an “interview stick” (Thomson 1988), an approximately 1.5‐m stick with a water‐filled flower pick attached to the end, into which a flower could be inserted and presented to foraging insects. After the visit, we removed the anthers from the flower into centrifuge tube with 1.00 mL 70% ethanol, being careful not to dislodge the remaining pollen grains. At the end of each day, we collected the anthers from remaining unvisited male‐phase flowers to serve as unvisited controls. Therefore, if pollen was lost passively from male‐phase flowers throughout the day, our unvisited controls represented counts of pollen after that loss has occurred. In the laboratory, we counted the number of pollen grains in each sample (both visited and unvisited) using a Coulter Multisizer 3 particle counter (Beckman Coulter Inc., Brea, CA). We prepared samples for counting by adding 0.9% saline, weighing the total sample, and then sonicating it to dislodge pollen grains from anthers. We counted four 1 mL subsamples using the particle counter. We then multiplied the mean of the four subsample counts by the weight of the total sample to estimate the number of pollen grains in the total sample.

To estimate the proportion of pollen that a given pollinator removes in a single visit, we compared the mean number of grains remaining after all single visits by that flower visitor group to the mean number of grains in unvisited controls. (Grains in an unvisited flower – grains remaining)/Grains in an unvisited flower) (as in Wilson and Thomson 1991).

### Single‐visit pollen deposition

To measure single‐visit pollen deposition of each visitor group, we counted the numbers of pollen grains deposited on stigmas during single visits by individual pollinators to previously unvisited female‐phase flowers. To prevent contamination by self‐pollen, we emasculated the flowers during the male phase the day before; as often as possible, we removed the anthers before anther dehiscence. We obtained visits to female‐phase flowers using the same methods as for the removal samples. After a visit, we collected the flower and placed it in a flower pick with water in a cooler for 24 h to prevent additional visits and to allow deposited pollen to adhere to the stigma and begin pollen tube growth. Then, we removed the stigma with forceps and placed it in a microcentrifuge tube filled with 70% ethanol for storage. In the laboratory, we mounted each stigma on a slide with fuchsin jelly and counted the number of pollen grains deposited.

### Visitation

To determine representative visitation rates to *C. virginica*, we conducted observations of specialist and generalist insect visitation to *C. virginica* male‐ and female‐phase flowers at all study locations during the daily activity window. We first determined a group of fresh flowers that we could observe simultaneously. We then observed this group for 5 min and counted visits by each visitor group. We conducted a set of visitation observations approximately every hour during the daily activity window.

### Depletion

We measured the pollen depletion rate during the daily activity window in five plant populations in suburban Philadelphia over 7 days in late April 2009; we processed a subset of these samples to generate ten site–date‐specific pollen depletion curves. On each site–date, we collected the anthers from a set of eight flowers in the population every hour. We collected anthers during the daily activity window; on most days, we collected two to three sets of anthers. Anthers from individual flowers were placed into separate microcentrifuge tubes (one tube per individual) filled with 1 mL of 70% ethanol. We chose flowers haphazardly, regardless of how many anthers had dehisced, and attempted to sample flowers from throughout the population during each collection period. We counted pollen following the same method as for the removal samples.

During the counting process, sonication opened and emptied undehisced anthers; therefore, our counts represent the full number of pollen grains in the flower, including those from anthers that had not yet dehisced at the time of collection. Therefore, pollen depletion in our data measures the number of grains remaining in the flower rather than pollen available to visiting pollinators.

### Data analysis

We compared the number of pollen grains removed and deposited by pollinator groups: the pollen‐specialist *A. erigeniae*, the bee‐fly *B. major*, and generalist bees in the genera *Lasioglossum*,* Ceratina*, and *Hylaeus*. We grouped these three bee genera into one functional group (“small generalist bees”). We compared the number of pollen grains deposited and the number of grains remaining in anthers after a single visit among visitor groups using generalized linear models (GLMs). For each, the predictor variable was the pollinator group (*A. erigeniae* females, *B. major*, or small generalist bees), and the response variable was the number of grains deposited or the number of grains remaining after a single visit by that pollinator. We used a negative binomial error distribution for both the removal and deposition models because the response variable in both data sets was overdispersed (Lindén and Mäntyniemi 2011). Analyses used R 3.0.1 (R Core Team 2013). For removal and deposition models, we used the R function *glm.nb* in the library *MASS* (Venables and Ripley 2002) and conducted multiple comparisons using the *glht* function in the library *MULTCOMP* (Hothorn et al. 2008).

To evaluate how the hour of collection affected the number of grains remaining in male‐phase flowers, we used generalized linear mixed models (GLMMs) with time since anthesis as the predictor and the total number of pollen grains in an open flower as the response variable. Because depletion could vary due to factors of the specific day of data collection, we included the day as a random effect. We used the function *glmmADMB* in the *glmmADMB* library (Fournier et al. 2012) because it allowed us to include random effects and account for overdispersion using a negative binomial distribution. We observed no autocorrelation structure in the average of the residuals over time.

## Results

### Single‐visit pollen removal

**Figure 2:**
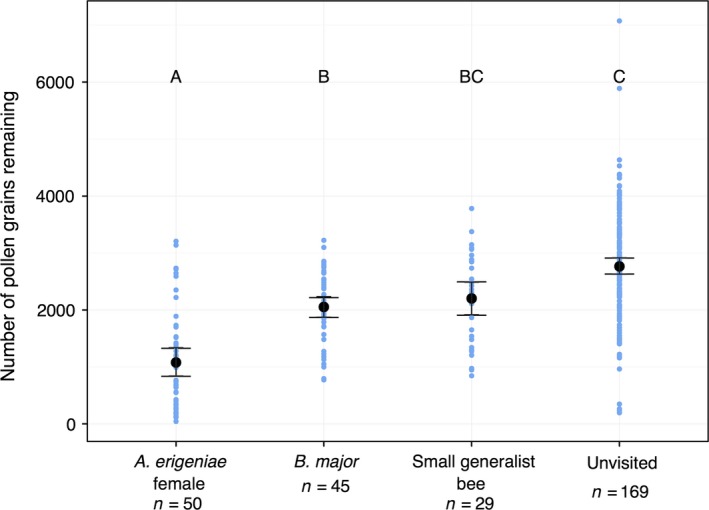
Plot of pollen grains remaining after a single visit to *C. virginica*. Small points are individual data points. Large points are means ± 95% CI. Boxes not sharing a letter are significantly different at *P *=* *0.05.

An individual male‐phase *C. virginica* flower contained on average 2764 ± 952 grains (mean ± SD). *Andrena erigeniae* females removed 61% of the pollen available in a single visit, more than *B. major* (23.7%, Table 1, Fig. 2, *Z* = 4.243, *P *<* *0.001) and small generalist bees (20.31%, Table 1, Fig. 2, GLM, *Z *=* *4.391, *P *<* *0.001).

**Table 1:** Measurements of visitation rate, single‐visit removal and deposition, and calculated pollen transfer efficiency for common flower visitors of *Claytonia virginica*. 2764 ± 952

Flower visitor	Visitation rate	Removal sample size	Number of grains remaining ± SD	Mean proportion removed, %	Number of grains removed	Deposition sample size	Number of grains deposited ± SD	Percent of grains removed that were deposited, %
*Andrena erigeniae female*	1.05 (to female) 2.10 (to male)	50	1078 ± 918	61	1686 ± 252	53	39.43 ± 52.07	2.33
*Bombylius major*	0.07 (to female) 0.03 (to male)	45	2053 ± 623	23.7	711 ± 720	22	30 ± 18.97	4.22
Small generalist bee	0.21 (to female) 0.45 (to male)	34	2203 ± 812	20.31	561 ± 497	30	14.97 ± 12.96	2.67

### Single‐visit pollen deposition

**Figure 3:**
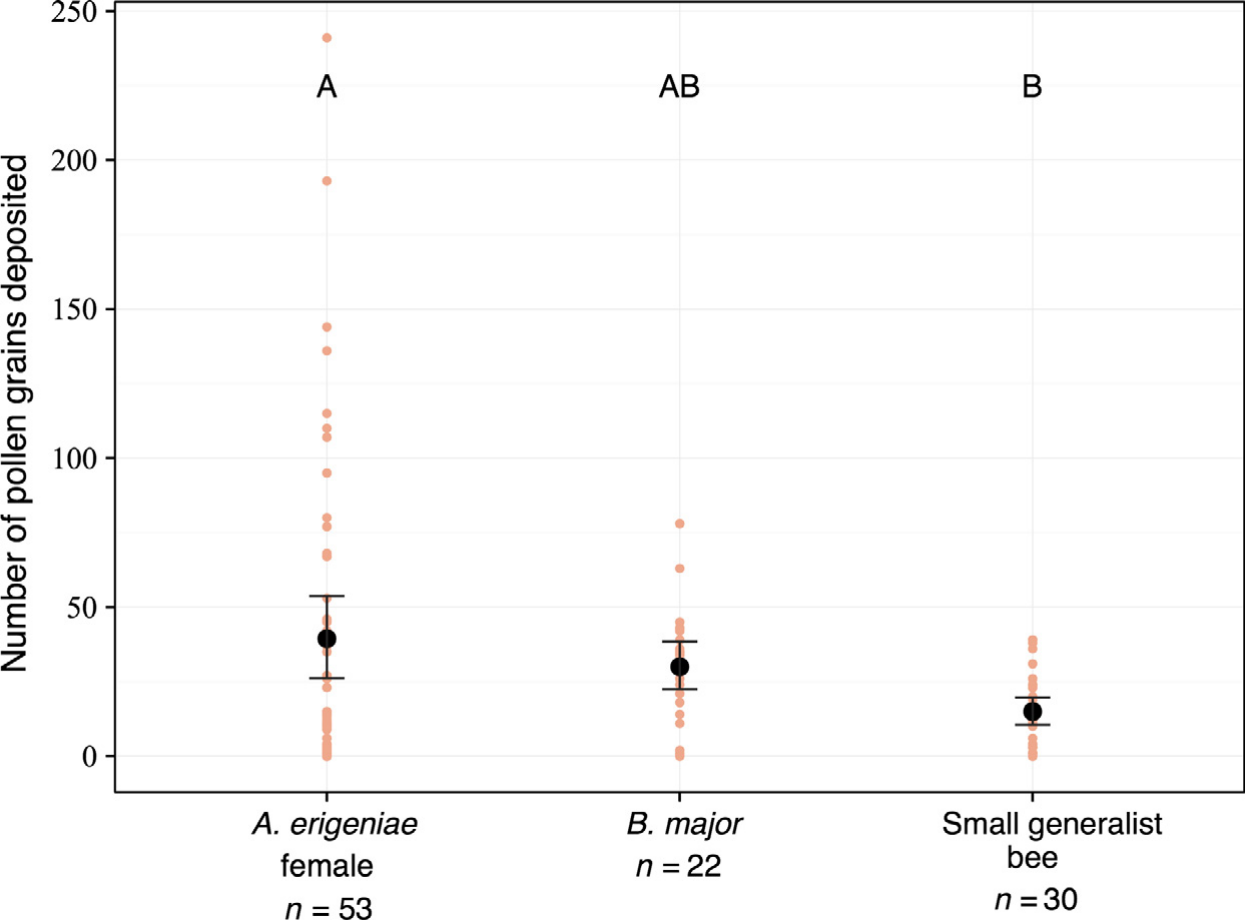
Plot of pollen grains deposited during a single visit to *C. virginica*. Small points are individual data points. Large points are means ± 95% CI. Boxes not sharing a letter are significantly different at *P *=* *0.05.

*Andrena erigeniae* females deposited more pollen in a single visit than small generalist bees deposited (39.43 ± 52.07 and 14.97 ± 12.96 grains, respectively, Table 1, Fig. 3, GLM, *Z* = 3.621, *P *=* *0.005). *Andrena erigeniae* deposited more pollen than *B. major*, but not significantly so (39.43 ± 52.07 and 30.00 ± 18.97 grains, respectively, Table 1, Fig. 3, GLM, *Z* = 0.928, *P *=* *0.98).

### Visitation

**Figure 4:**
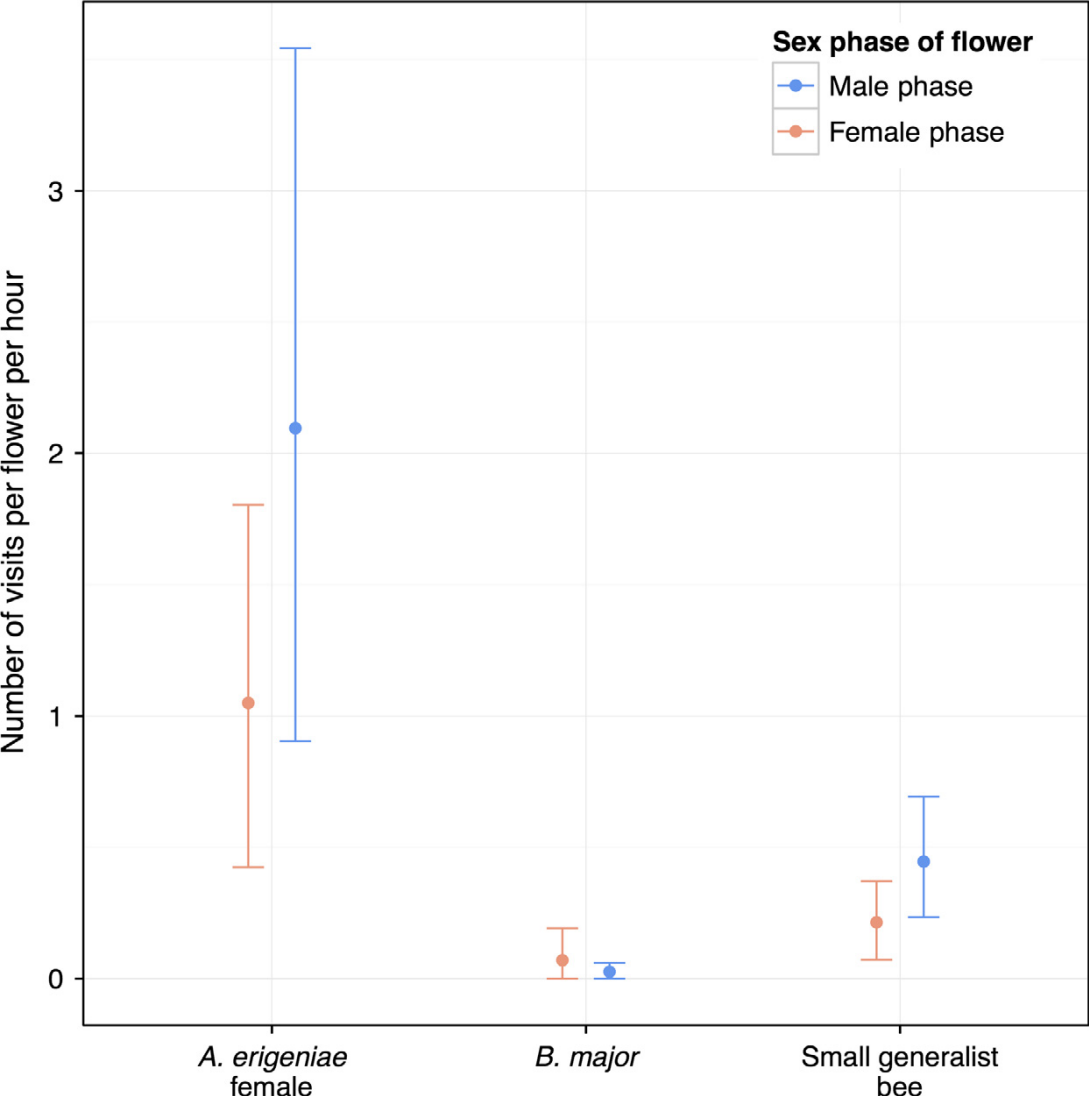
Visitation rates by *C. virginica* flower visitors in Pennsylvania. Rates are means ± 95% CI.

With a visitation rate of over ten times higher than *B. major* and five times higher than small generalist bees, *A. erigeniae* were by far the most common visitor (Table 1, Fig. 4). Both *A. erigeniae* females and small generalist bees showed a preference for male‐phase flowers, visiting male phase approximately twice as often as female‐phase flowers (Table 1, Fig. 4). Insect activity began as flowers opened. *Andrena erigeniae* females visited more often in the morning hours than in the afternoon hours, slowing as floral rewards were depleted. For example, the average *A. erigeniae* visitation rate from 9 to 10 am (2.42 visits per flower per hour) was much higher than the average visitation rate from 12 to 1 pm (0.91 visits per flower per hour). *Bombylius major* visitation was consistent throughout the day and continued into the afternoon, well after pollen was depleted.

### Depletion

**Figure 5:**
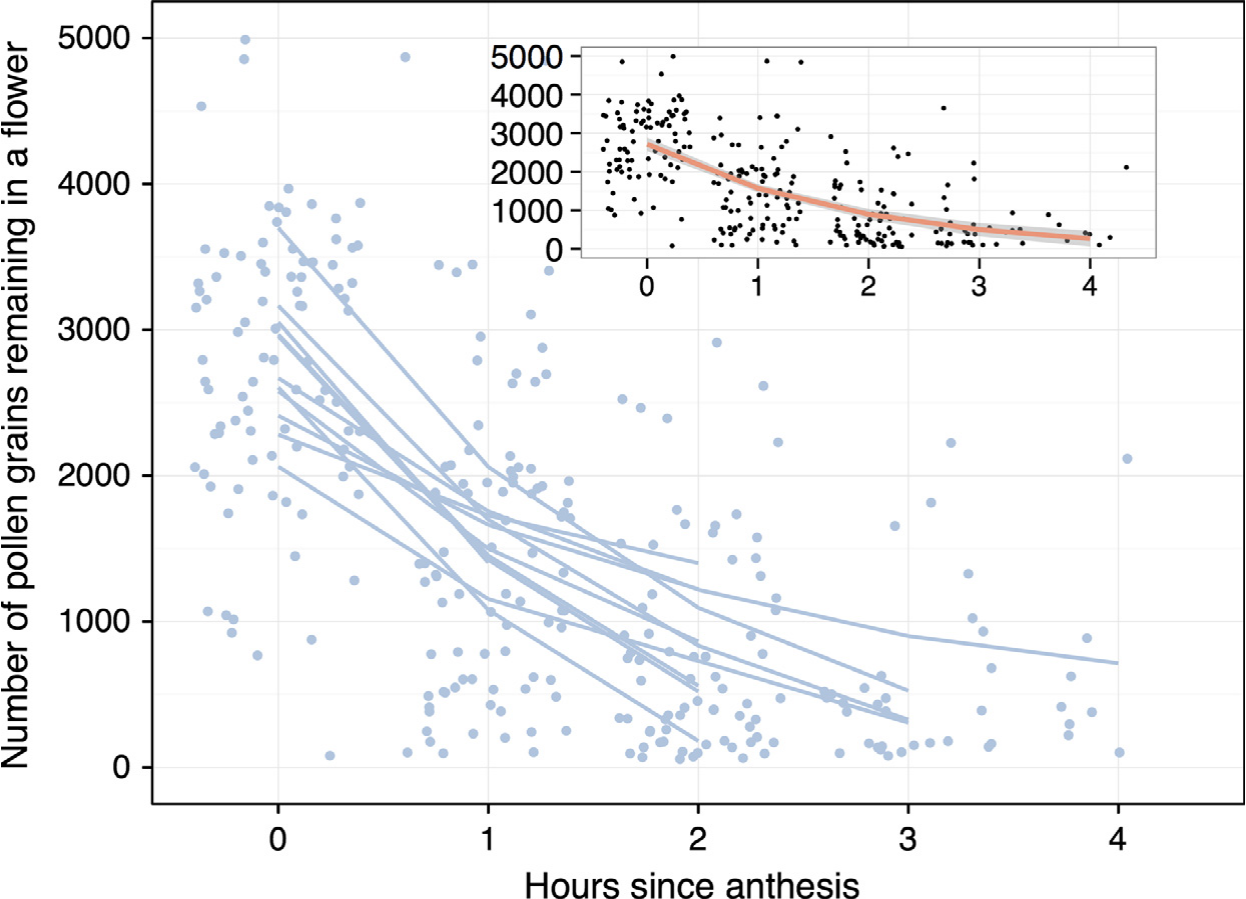
Regression of pollen depletion over time. (i) Points are actual measurements; lines are our statistical model fit to each individual day. (ii) The pink line in the subplot is the fit of the statistical model.

Pollen depletion is rapid in these *C. virginica* populations. In the first hour, the mean number of grains per flower was 2761 grains, which dropped to 1509 grains in the second hour and 804 grains in the third hour. Including the time since anthesis significantly improved the model fit (Fig. 5, GLMM, *r* ^2^ = 0.34, *Z* = −12.1, *P *<* *2e‐16).

## Discussion

From our measured values of visitation and pollen transfer, there appear to be substantial costs and substantial benefits to *C. virginica* from pollen‐specialist *A. erigeniae* female visitation relative to the other flower visitors. In Pennsylvania populations of *C. virginica*,* A. erigeniae* females visited four times as much as all other visitors combined. *Andrena erigeniae* females also visited male‐phase flowers approximately twice as much as female‐phase flowers (1.05 visits and 2.10 visits per hour, respectively). In a single visit, an *A. erigeniae* female removed on average 61% of the pollen on a male‐phase flower. *Andrena erigeniae* females monopolize the pollen supply through high visitation rates to male‐phase flowers and high single‐visit removal values relative to the other flower visitors. In these populations of *C. virginica*, pollen is depleted quickly. Our observed pollen depletion is the result of visitation by the whole community of flower visitors, rather than *A. erigeniae* females only. However, our evidence suggests that the pollen specialist is the primary driver of the observed pollen depletion. *Andrena erigeniae* visit earlier; *A. erigeniae's* highest average visitation rate occurred between 9:00 and 10:00 am while during the same time period, there was no visitation at all by *B. major* in our data set. *Andrena erigeniae* also has a substantial contribution to pollen deposition. Although these bees deposit an unremarkable quantity of grains per visit in comparison with other pollinators (not significantly different from *B. major*), their mutualistic contribution is multiplied through numerous female‐phase visits.

In this system, the cost of pollination service by nectar‐collecting flies and the small generalist bees is minimal. Neither group removed nearly as many grains as *A. erigeniae* females; for the small generalist bees, this may indicate that they were collecting mostly nectar from *A. erigeniae* or that their small size prevented substantial pollen collection. The pollen‐collecting generalist bees do not deposit as many grains as *A. erigeniae* females in single visits; combined with their relatively low visitation rates and low removal rates, these pollen‐collecting generalist species do not seem to have a substantial impact – either positive or negative – on *C. virginica* pollination in these populations. *Bombylius major*'s contribution to pollen deposition is as high as *A. erigeniae* females' on a per‐visit basis, but does not result in high absolute deposition because their visitation rates are so low.

*Claytonia virginica* pollen is depleted quickly. The only other studies that measure pollen depletion found similarly extreme rates of pollen depletion in 1 day of visitation (Wilson and Thomson 1991; Minckley et al. 1994; Stanghellini et al. 2002; Raine et al. 2006; Raine and Chittka 2008). Moreover, our measurements of pollen depletion included grains in as‐yet undehisced anthers; we measured the number of grains *remaining* in a male‐phase flower, rather than the number of grains *available*. *Claytonia virginica*'s gradual anther dehiscence seems to delay pollen depletion because pollen cannot be removed until it is made available. The rate of pollen depletion would be even more extreme if all of the pollen in a male‐phase flower was presented at once; this is evidence that the packaging of pollen into ten units, and the gradual dehiscence of these units, may be a response to selection to limit *A. erigeniae* pollen overexploitation. There is evidence that high rates of pollen collection and depletion may select for sequential and slow pollen dehiscence; pollen packaging schedules have been shown to vary with the pollination context in three *Epimedium* species (Li et al. 2014).

Visits by *A. erigeniae* females may – in some cases – be more detrimental than beneficial within the context of these Pennsylvania *C. virginica* populations. On average, *A. erigeniae* female bees remove over one thousand pollen grains in a single visit to a male‐phase *C. virginica* flower and then deliver only 2.33% of those grains; many of the remaining grains probably provision bee offspring and are wasted from the plant perspective. *Andrena erigeniae* females also make approximately two male‐phase visits for every female‐phase visit. Thus, because of *A. erigeniae* pollen collection, the male function of *C. virginica* individuals is reduced. If the wasted grains could have been delivered to *C. virginica* stigmas by a subsequent pollinator, then visits by *A. erigeniae* females would be lowering overall pollen delivery in *C. virginica* populations. Moreover, if the populations are pollen‐limited, then lowering overall pollen delivery may also be lowering overall seed production. In these populations, there is the potential that wasted grains could have been delivered to *C. virginica* stigmas by a subsequent visitor because a diversity of other flower visitors was present, visited with high frequency, and has the potential to contribute substantially to *C. virginica* pollination if their numbers increased. In our system, the best candidate for this role is *B. major*, which delivered similar numbers of pollen grains to *C. virginica* female‐phase flowers but with less pollen wastage (delivering 4.22% of the pollen it removed).

Pollination relationships are complex, and other factors will be important in determining pollinator value to plants. Pollinator type and pollen removal by bees may affect the selfing and outcrossing rate (Brunet and Holmquist 2009), pollinator groups vary in the relative amount of conspecific pollen carried on their bodies (Alarcón 2010), and the value of floral visitors to plants may depend on foraging behavior within taxa (Young et al. 2007). Also, competitive interactions may be important; *Andrena erigeniae* females may be competitively excluding other flower visitors by monopolizing *C. virginica* nectar and pollen, meaning that a decrease in *A. erigeniae* visits could increase visits by other flower visitors. If *A. erigeniae* were not monopolizing floral resources, would *B. major* visitation rates increase? Would more *C. virginica* pollen be delivered to female‐phase flowers? Evaluating floral constancy, variation in the pollinator community, and the effects of competitive interactions are important in order to fully understand the nature of these relationships.

The degree of mutualism between *A. erigeniae* and *C. virginica* is likely to change with changes in the pollinator context, and *C. virginica* is likely to face a different pollinator context with differences in geography or phenology. Insect populations are known to drastically fluctuate, so *A. erigeniae*,* B. major*, and small generalist bee populations may vary stochastically, or with changes in geographic, climatic, and seasonal patterns. For example, Motten et al. (1981) report much higher visitation by *B. major* in North Carolina populations than we report for Pennsylvania populations. These kinds of changes to the pollinator context may change the value of *A. erigeniae* females to *C. virginica* because of the potential effect on the effectiveness of subsequent pollinators. Therefore, despite transferring large amounts of pollen as a pollinator, *A. erigeniae* may vary in the benefits that it provides to *C. virginica* pollination.

## Conflict of Interest

None declared.
